# Promoting and regulating generic medicines: Brazil in comparative perspective

**DOI:** 10.26633/RPSP.2017.5

**Published:** 2017-02-08

**Authors:** Elize Massard da Fonseca, Kenneth C Shadlen

**Affiliations:** 1 Institute of Education and Research Institute of Education and Research São Paulo Brazil Institute of Education and Research, São Paulo, Brazil.; 2 London School of Economics and Political Science Department of International Development London United Kingdom London School of Economics and Political Science, Department of International Development, London, United Kingdom.

**Keywords:** Drugs, generic, therapeutic equivalency, generic drug policy, Brazil, Medicamentos genéricos, equivalencia terapéutica, política de medicamentos genéricos, Brasil

## Abstract

Promoting the use of generic drugs can constitute a core instrument for countries’ national pharmaceutical policies, one that reduces drug expenditure while expanding health care access. Despite the potential importance of such policy measures and the differences among national practices, scholars embarking on comparative analysis lack a roadmap for determining which dimensions of generic drug policy to assess and compare. This report fills that gap by considering national rules and regulations across four dimensions deemed crucial to any evaluation: demonstrated therapeutic equivalence; pharmaceutical packaging and labeling; drug prescription; and drug substitution. Furthermore, this report examines how the diverse interests of public and private sector stakeholders might shape generic drug policy and its implementation. To illustrate the challenges and conflicts behind policy development and implementation, this report focuses on the case of Brazil.

## RESUMEN

La promoción del uso de medicamentos genéricos puede ser un instrumento fundamental para la política farmacéutica nacional de los países, puesto que reduce el gasto en medicamentos y, a la vez, amplía el acceso a la atención de salud. A pesar de la importancia que pueden tener esas políticas y de las diferencias entre las prácticas de cada país, los expertos que emprenden análisis comparativos carecen de una hoja de ruta que les permita determinar los aspectos que deben evaluar y comparar de las políticas relativas a los medicamentos genéricos. Este informe subsana esta carencia al examinar las leyes y los reglamentos nacionales en relación con cuatro dimensiones consideradas cruciales en toda evaluación: la demostración de la equivalencia terapéutica, el empaquetado y el etiquetado farmacéutico, la prescripción, y la sustitución. Además, se examina la forma en que los distintos intereses de las partes involucradas de los sectores público y privado podrían configurar la política relativa a los medicamentos genéricos y su aplicación. A fin de ilustrar los retos y los conflictos que subyacen a la formulación y la ejecución de las políticas farmacéuticas, este informe se centra en el caso del Brasil

To reduce the price of prescription medications, the World Health Organization (WHO) recommends that generic drug substitution be a component of every country’s National Pharmaceutical Policy (1). To that end, WHO established guidelines for exchanging products, technical criteria to determine when one pharmaceutical product can be safely and effectively used in the place of another, as well as policies to promote a higher rate of generic drug use (2).

A great deal is known about the various policy instruments and mechanisms that countries can use to promote the demand for and supply of generic drug products (1–3). And, anecdotal evidence and casual observation suggest that there is significant variation in national pharmaceutical policies with regard to generics (4, 5). A challenge to comparative analysis, however, is the lack of agreement, especially on which particular dimensions of policy are relevant for understanding generic drug promotion and supply. This paper advances a taxonomy of generic drug substitution systems that can be used for such an analysis. To illustrate its utility, the study applies the proposed taxonomy to the case of Brazil. Our taxonomy refers to regulation of chemically derived generic product. Regulation of biosimilar medicines–that is a product of biological origin, usually with a complex structure, and large proteins–engage with different conflicts and challenges.

## DIMENSIONS OF ANALYSIS

To understand the diversity of national practices regarding promotion and regulation of generic medicines, certain key questions must be considered. For example, how have generic drug products been demonstrated to be therapeutically equivalent to originator products (equivalence)? Are generic products allowed to display brand names (labeling)? Should doctors prescribe using the generic name or can they use a brand name (prescription)? Are pharmacists authorized to substitute a generic product for an innovator product (substitution)? These four dimensions are crucial to any evaluation i.e.: demonstrated therapeutic equivalence; pharmaceutical packaging and labeling; drug prescription; and drug substitution. The significance of these dimensions are explored below.

### Equivalence

Regulatory authorities set standards that determine which drugs need to be therapeutically equal to reference products, and how such equivalence is demonstrated. For drugs that need to demonstrate equivalency, this requires tests of bioavailability (BA) and bioequivalence(BE). BA measures the extent to which a drug is absorbed into the body and is available to act upon its intended target (the site of action). BE considers whether or not there is any significant difference in the rate and extent of availability of two drugs over a period of time, at the same dose and under the same conditions. Follow-on products that have the same BA as the reference product are regarded as “generic” products, while those with differing BA are not (6).

Demonstrating BE is essential for generic drugs. A small difference in bioavailability may alter a drug’s effect, and if so, cannot be considered equivalent. The establishment of BE is particularly crucial when considering medicines with highly toxic ingredients or in a Narrow Therapeutic Range (NTR),^3^ i.e., where small differences in dosage can have toxic effects. National regulatory authorities have discretion to define how to measure a product’s NTR, and also, to determine which medicines require BE testing (7). These are decisions each country must make for itself. BE has arguably been associated with quality control (8), and determining which products need to undergo it has often been challenging (9)._4_

### Labeling and packaging

The second dimension for comparison pertains to a country’s regulations regarding labeling and packaging of generic products. Use of the generic name or international non-proprietary name (INN), usually a simplified version of the chemical name, can remove the obscurities that brand names create. Not only may the INN be displayed on the pharmaceutical packaging, but also font size and presentation will differ according to local regulations. For instance, some countries require the INN to be no less than 30% – 50% smaller than the font size of the brand name; some require that it be of equal size; while others still have banned the .use of brand names altogether (1). Regulation of pharmaceutical packages and brands is very important since marketing strategies represent an important element of the product cycle (10). As further discussed below, this is true not just for “innovator” firms, but also for follow-on “generic” producers.

### Prescription and substitution

Prescription and substitution are the third and fourth dimensions of this taxonomy. The use of the generic name facilitates the prescription and dispensing of pharmaceuticals to patients, as well as communication among health professionals and scientists (11). It also allows for easy “comparison shopping,” as there might be different suppliers of the same pharmaceutical product. Depending on national regulations, physicians may be required to prescribe by generic name; or they may include the brand name and recommend that it be supplied; or they may forbid substitution. Other regulations may permit pharmacists to consult the patients as to whether or not they prefer the brand name medicine or the generic.

This discussion of prescription and regulation is, of course, closely tied to the previous discussion of labeling and packaging. After all, pharmaceutical firms invest heavily in distinguishing their brands, and actively promote their brands among physicians, pharmacists, and patients. These promotional efforts can create incentives to prescribe or substitute one product for another. Even if health professionals have no doubts about the quality of generic medicines, they may be disinclined to prescribe them, as may pharmacists (12).

## APPLICATION OF THE TAXONOMY

Through the lens of the proposed taxonomy, the study examined the generic drug policies of Brazil. Findings were based on empirical data collected in 2007 – 2015, including national government documents (e.g., policy memos, official speeches, etc.) and more than 400 newspaper articles and scientific papers. These data were supplemented by 60 interviews with key informants who had participated in designing and implementing Brazil’s generic drug policies, e.g., regulators, government officials, and representatives of local and multinational pharmaceutical companies.

### Generic substitution in Brazil

Brazil is a case study that is crucial to understanding the regulation of interchangeable pharmaceutical products. Among Latin American countries, Brazil has the largest generic drug sector, representing almost 28% of the country’s pharmaceutical sales. While Brazil has witnessed high levels of generic market penetration, the process has been accompanied by a number of conflicts and challenges.

### Equivalence

In 1999, the Ministry of Health of Brazil took a decisive step in promoting generic equivalents by enacting the Generic Drug Act (13). This legislation required demonstration of BE as a condition for market entry._5_ It also promoted a major reform of the parameters for registering off-patent pharmaceutical products in Brazil. The introduction of rules governing therapeutic equivalence represented one of the most contentious elements of the legislation, affecting the country’s pharmaceutical sector and highlighting political controversies surrounding drug substitution.

Brazil requirements are relatively stringent compared to those of other Latin American countries. A study conducted by the Pan American Health Organization (PAHO) concluded that of the 86 drugs analyzed in Latin American countries, 51 required demonstration of bioequivalence in Brazil (11). No other national regulatory authority examined by that study requested bioequivalence for so many drugs (14). Many local pharmaceutical firms claimed they would be unable to comply, given the high costs and complications of testing and a lack of expertise in Brazil. In response, the country’s regulatory authority took decisive steps of supporting and advocating for bioequivalence tests and fostering close collaboration with local industries to help national producers meet the new requirements (15). For instance, the regulatory authority created a fast track approval process for firms prepared to register generic products. It also provided continuous consultation and support to local firms by clarifying and supervising changes to their regulatory departments. Whereas in 2002 only 27.3% of BE studies were conducted in Brazil, by end of 2009, 87.6% were performed in the country (16).

Many local firms not only managed to adapt to the new requirements, but became market leaders in the pharmaceutical sector (15). The firms that adapted to the new regulations soon saw generic drugs as a valuable opportunity in terms of market share and improved capability. Local pharmaceutical companies in Brazil now account for 88% of the domestic generic drugs market. Table 1, based on IMS Health data (1999 and 2001–2011) that includes both patent and off-patent products collected from the retail market only (excluding government data for primarily essential medicines, AIDS treat-ment, and other patented/high cost products) demonstrates the progressive growth of the pharmaceutical sector and the current-day status of local firms.

Thanks to the gains in industry capabilities brought on by the BE resolution, and the increased importance of local producers, the national pharmaceutical sector became a policy priority. Pharmaceutical sector representatives assisted the Government of Brazil in identifying bottlenecks to the sector’s expansion (17). Since Brazil is highly dependent on the importation of key inputs for medicine production, e.g., raw materials and active pharmaceutical ingredients, this area was identified as an investment priority (17, 18). The consensus among representatives of the pharmaceutical sector is now that the generic drug regulations, first seen as a threat to their survival, were ultimately instrumental in improving drug manufacturing plants and processes.

### Labeling and packaging

The regulation of labeling of pharmaceutical products is not a recent issue in Brazil. Three attempts to regulate packaging and non-proprietary names have raised heated political debates.**First, in the early 1990s, an unsuccessful Congressional initiative (PL 2022/1991) proposed banning the use of brand names from all pharmaceutical products. At that time, there were two types of products in the market: reference products (usually the innovator products) and similar medicines (copies of the reference product, but without equivalence tests), both types commercialized under their respective brand names. This proposal, which was motivated by the fact that 50 million people had limited access to medicines, would have required all pharmaceutical products in Brazil to be prescribed and commercialized using the INN, or in cases where INN was not available, the Brazilian non-proprietary name (BNN). Expecting that a reduction in the use of brand names would lower costs and facilitate interchangeability, the proposal would have only allowed brand names to be included on packages if they were presented in a font size smaller than the generic name; all public health service prescriptions would use the generic name.

In 1993, and concurrent to Congressional negotiations, the Ministry of Health promoted a second attempt to regulate the labeling and packaging of generic drugs. The Ministry of Health sponsored Presidential Decree 793/1993, which required, among others, that the fonts used for brand names be no greater than one-third the size of the generic name, and that all drugs prescribed and procured by the National Health System use generic names. The pharmaceutical industries and drug retailers promptly reacted through the courts, arguing these requirements would harm their businesses (19), and neither the Congressional initiative nor the Executive Decree survived (20).

It was only with the passage of the Generic Drug Law in 1999 that discussions on INN and labeling progressed. The Law also stipulates that all generic drugs provide only the INN, and that packaging include a yellow stripe with the letter “G” to indicate the product is interchangeable. In contrast, for the labeling of innovator products, the trademark can be displayed in a larger font size, with the INN or BNN immediately below, in a font no less than half the size of the brand name. The packaging of similar drugs would have the same regulatory standards as the innovator products, but would not be interchangeable because, unlike generic products, they did not have equivalence test results available.^6^

**TABLE 1.** Ranking of pharmaceutical companies in Brazil (US$), 1999 and 2001–2011

Company	Year													2011 market participation (%)
	1999	2000	2001	2002	2003	2004	2005	2006	2007	2008	2009	2010	2011	
EMS (Brazil)	29	–^a^	12	6	5	5	5	3	2	1	1	1	1	7.77
Medley (Brazil)^b^	32	–	19	12	7	6	7	6	4	4	4	2	2	7.11
Ache (Brazil)	3	–	2	2	2	2	2	2	3	3	3	4	3	5.24
Sanofi (France)	1	–	1	1	1	1	1	1	2	2	2	3	4	4.63
Eurofarma (Brazil)	28	–	25	21	19	16	9	8	6	6	6	5	5	4.14
Neoquimica (Brazil)	–	–	48	48	39	36	39	38	36	31	20	8	6	3.71
Novartis (Switzerland)	2	–	4	4	4	4	4	4	5	5	5	6	7	3.54
Merck Sharpe Dome (United States)	–	–	9	7	8	10	16	17	7	8	8	9	8	2.56
Pfizer (United States)	7	–	3	3	3	3	3	5	6	7	7	7	9	2.43
Bayer (Germany)	23	–	16	12	17	11	6	7	7	8	8	11	10	2.16
AstraZeneca (United Kingdom)	19	–	21	22	23	23	22	20	15	12	9	10	11	2.03
Teuto (Brazil)	–	–	37	39	48	50	54	50	43	38	29	16	13	1.89

Source: Prepared with information from Pro-Genericos (30) and Sindusfarma (São Paulo, Brazil).

a Data unavailable.

b Medley (Brazil) was purchased by Sanofi (France) in 2005.

### Prescription and substitution

During the debates in Congress that led to the Generic Drug Law, the prescription rules for physicians were highly controversial. The Government and national and multi-national pharmaceutical industries, all disagreed starkly on this component of the bill. The multinational pharmaceutical industry demanded that generic drug substitution only be allowed by a doctor’s written request. However, the Government did not agree to negotiate this aspect of the bill; thus, if doctors do not agree with substitution, they must indicate “substitution not allowed” on the prescription (21). While doctors in the National Health System are obligated to prescribe using the generic name, private physicians are not bound by this rule, and thus can continue to prescribe by brand name.

### Effects and emerging challenges in Brazil

The prescription of generic medicines by INN is still low, but has increased over time, representing 20.9% of total prescriptions in 2006, compared to 11.8% in 2002 (22) (22). Despite the growth of the generic drug market in Brazil, there is still low consumer awareness regarding drug substitution and slow acceptance by physicians (23). Studies suggest that there is confusion on how to differentiate between pharmaceutical products (innovator, similar, and generic) and a lack of confidence in the quality of generic drugs (24, 25).

In terms of generic drug prescription, academic studies, market assessments and a number of newspaper articles point out that health professionals still resist prescribing generic drugs (26, 27). For example, a survey conducted in 2006 in eight Brazilian cities assessed the opinion of 55 health professionals. Results showed that 44% of the health professionals believed that generic drugs were not as reliable as the originals, and that even among those who trusted generic drugs, 17% did not prescribe them (28).

### Recent reforms and challenges in Brazil

In response to the lack of confidence in generic and similar medicines among public and health professional, in 2014 the Government of Brazil proposed new regulation to clarify which pharmaceutical products are therapeutically equivalent. At the suggestion of the Ministry of Health, the National Regulatory Agency (ANVISA) proposed a new resolution to modify the packaging of pharmaceutical products (29). The new resolution would allow pharmacists to substitute the reference product for a generic or similar product.

To understand the challenges facing health policymakers in Brazil, keep in mind that, in contrast to the situation prior to the launch of the generics policy, most non-originator drugs have demonstrated bioequivalence. Most similar drugs are now BE; very few drugs that have not demonstrated BE remain on the market. Yet most of these non-originator BE drugs continue to have brand names. These branded BE drugs, essentially like “branded generics” commercialized in retail markets in the United States of America and the United Kingdom, represent 47% of the pharmaceutical market (units), while formally “generic” drugs (i.e., BE and without a brand) represent 27% (Table 2). Yet, substitution is only allowed for generics; similar drugs cannot be exchanged once prescribed by a doctor. The Government’s intention was to add the symbol “EQ”—a visual label meaning a product can be switched for another—to the packaging of inter-changeable products.

In suggesting the use of “EQ,” health authorities argued that this would increase consumer options among products with proven therapeutic equivalence, thereby reducing price. The Government maintains that this regulation is a logical follow-up and response to Resolutions 133 and 134 passed in 2001 that established the year 2014 as the deadline for similar medicines to submit bioequivalent testing for agency approval. Different from the discussion in the early 2000s that centered on quality and manufacturing processes, the EQ debate was only concerned with the labeling of pharmaceuticals. The announcement was made in January 2014 by the Minister of Health and this sparked heated debate among pharmaceutical industry representatives. Technically, they argued, it was reasonable, since all products are the same and have the same active ingredients and therapeutic responses (per-sonal communication, Chief Executive Officer of a Brazilian pharmaceutical company, February 2014). However, the EQ label would commodify reference products and similar medicines. Because these are brand-name products, they rely on strong marketing strategies.

Therefore, pharmaceutical firms—national and multinationals–that sell their products under brand names feared that they would be adversely affected by this regulation, that the presence of EQ on the label would essentially send a message to ignore brand markings. After much discussion, a resolution was issued in October 2014, responding to the demands of the pharmaceutical industries: no EQ symbol would be added to labels, but rather, the leaflets found inside of pharmaceutical packaging would indicate if the product was interchangeable. As a result of this compromise, however, this information will not be available until the product had already been purchased and opened.

**TABLE 2.** Distribution of pharmaceutical products by value (R$^a^) and units in Brazil, August 2013–July 2014

Pharmaceutical product	Value (R$)	Units
Similar drugs	44.48%	47.75%
Similar drugs (without BE^b^)	0.39%	0.57%
Reference product	30.83%	23.81%
Generic drug	24.29%	27.86%
Total	62 132 559 369	3 010 750 992

Source: IMS Health. Data on pharmaceutical sector in Brazil from personal communication with IMS representative (email, 29 August 2014).

a Mean of exchange rate R$ 1 : US$ 2.29 in August 2013–July 2014.

b Bioequivalence.

The debate over the EQ resolution is important for two reasons. The resolution intended to diminish the role of branding by emphasizing the equivalence of equivalent products. In doing so it would increase the scope of substitution, and it was expected, reduce the price of drugs. Although the idea behind the EQ proposal has a strong public health rationale, the structure of the pharmaceutical market in Brazil creates economic interests that were able to di-lute the measure—and may yet subvert this policy instrument. The debate also illustrates how Brazil innovates in generic regulation, not just using traditional instruments of interchangeability (i.e., the INN), but with additional information in the package leaflet.

### Conclusions

This analysis draws attention to the various dimensions of national generic drug regulations and their core policy instruments; it contributes to the literature by building new conceptual and empirical evidence on developing countries’ compliance with generic drug guidelines. To understand regional differences and regulatory choices, one must clarify the incentives and public health interests of these instruments and the institutional marketing opportunities.

In the Brazil case, these relationships were demonstrated through the regulation of INN and prescription rules, and bioequivalence and pharmaceutical packaging. The case of Brazil also demonstrates that these are not just technical concepts, but rather, highly contested political decisions. The Generic Drug Law of 1999 was an opportunity to foster the use of the INN in Brazil as a prescription rule and improve the pharmacology requirements to register non-patent drugs. This paper also pointed to the strong political conflicts that can be generated by efforts to apply new regulations regarding generic drug instruments.

Taking a step back, core lessons and implications from applying the proposed taxonomy to the Brazil case study are twofold. First, the diverse interests of the public and private sectors stakeholders shape the design and implementation of the core dimensions of any national generic drug regulation. Regulations that are effective and long lasting are designed with an understanding of the politics of drug substitution, i.e., their effects on public health, business concerns, and strategies. And secondly, that the task ahead is to think more clearly about the set of dimensions that influence national generic drug systems. This paper provides an initial step that is expected to encourage other scholars to evaluate, refine and apply the learnings in other contexts.

### Acknowledgements.

EMF receives funding from the São Paulo State Research Foundation (Grant No 2014/07725-3 and 2015/18604-5).

### Disclaimer.

Authors hold sole responsibility for the views expressed in the manuscript, which may not necessarily reflect the opinion or policy of the *RPSP/PAJPH* and/or PAHO.
